# The influence of mortality reminders on cultural in‐group versus out‐group takeaway food safety perceptions during the COVID‐19 pandemic

**DOI:** 10.1111/jasp.12740

**Published:** 2021-01-26

**Authors:** Simon McCabe, Seda Erdem

**Affiliations:** ^1^ University of Stirling Stirling Scotland

## Abstract

During the early stages of the COVID‐19 pandemic takeaway food orders generally increased, yet sales of Chinese and Italian food declined. At this time, news sources ran stories on the safety of cuisine from these countries, frequently juxtaposed with communications on mortality‐related information related to the virus. Terror management theory suggests mortality concerns can lead people to defend against the psychological threat of death by bolstering positive evaluations of products and values of their own culture, and by disparaging products and values of other cultures. This translates to food preferences, with death reminders heightening consumption of food from one's own (vs. others’) culture. However, whether this extends to food safety perceptions has not yet been probed. In the present experimental study, we examine whether death reminders (vs. a control topic) led U.S. participants to view American takeaway food as safer to consume, relative to Chinese and Italian food. Results indicate that across conditions, American food was evaluated as safer relative to Chinese and Italian takeout. Further, American takeaway was seen as safer after mortality reminders (vs. a control topic), with no differences in safety evaluations for Chinese or Italian takeout. Results are discussed in relation to the COVID‐19 pandemic.

## 1 INTRODUCTION

During the early stages of the COVID‐19 pandemic, interest in visiting restaurants broadly decreased around the globe, with data indicating many consumers switching to online delivery for grocery and takeout orders (e.g., Shakespeare, 2020). While many takeaway businesses benefitted from this migration to online food orders and takeout, news reports noted some vendors were missing out. Google search data, for instance, noted that searches for topics such as “Chinese restaurants” and “Chinese delivery” decreased substantially worldwide, and in the United States, interest in Chinese cuisine dropped by 17% (Williams, 2020). Similarly, Federalimentare, Italy's food & drink association, claimed there were a number of false and negative myths circulating about the safety of Italian food products. These changes in patterns of food consumption occurred against a backdrop of a worldwide pandemic that bombarded people with the reality of their own mortality. News reports at the start of 2020 provided constant updates from China and Italy, where the virus first dramatically took hold. These reports often included statistics and imagery related to the mounting death tolls, and government communications made clear that the threat of death was a possible outcome from contracting the virus. While on the surface concerns about mortality may appear unrelated to people's food preferences, one theory developed to understand how people might manage concerns about death—terror management theory (TMT; Greenberg et al., 1986)—argues people's responses to such existential threats can manifest in unexpected ways that may have implications for food safety concerns.

## 2 TERROR MANAGEMENT THEORY

TMT draws from the writings of Ernest Becker (1973) and proposes that the uniquely human awareness of death engenders potentially debilitating existential terror that is managed by embracing cultural worldviews that afford a sense of meaning and value in pursuit of literal and/or symbolic immortality. Further, TMT posits humans are motivated to manage the awareness of death via two distinct motivational orientations that are engaged dependent on the level of awareness of mortality (Pyszczynski et al., 1999). According to the theory, when death‐related thought enters consciousness, people become motivated to remove these cognitions from focal awareness, either by suppressing death‐thought or by denying, trivializing, or proactively reducing real or perceived vulnerabilities. In comparison, when death thoughts are active but outside conscious awareness, people become motivated to cultivate a sense of permanence by viewing themselves as having value (self‐esteem) within their seemingly permanent cultural system (cultural worldview), which they are driven to protect (often referred to as “worldview defense”).

## 3 MORTALITY SALIENCE, WORLDVIEW DEFENSE, AND PRODUCT/FOOD PREFERENCES

Early studies utilizing the terror management framework focused on more direct manifestations of worldview defense. As just a couple of examples, reminders of death (vs. control topics) have been found to lead judges to deliver harsher punishments for those who violated the law (Rosenblatt et al., 1989), and Christian participants evaluated fellow Christians more positively after death reminders, but evaluated Jewish people less favorably (Greenberg et al., 1990). More recently, studies suggest worldview defense can manifest in subtler ways, leading those who are reminded of mortality to defend against existential concerns by championing their own worldview and derogating other's worldviews via more indirect means. One such method through which worldviews can be supported or derogated is through consumer behavior. One striking illustration of this might be gleaned from examining the tragedy of 9/11. This terrorist attack could be construed as a mass mortality reminder for the U.S. population, and many Americans appear to have significantly increased the consumption of U.S.‐made goods versus foreign‐made goods following the event (United States Department of Commerce, 2008). Studies motivated by this pattern utilized the terror management framework to shed light on this phenomenon, suggesting the boost in sales of domestic products may have been driven, in part, by psychological strategies deployed to curb existential anxieties. Studies show, for example, that when U.S. participants viewed films of fatal car crashes, the participants who were reminded of their mortality blamed the car manufacturer more for the crash if the manufacturer was Japanese as opposed to American, presumably as a way to support their own worldview and derogate others (Nelson et al., 1997). Related to these findings, Jonas, Fritsche, and Greenberg (2005) asked German participants several questions referring to preferences for diverse cultural items (cars, cuisine, preferred European capital, etc.). They similarly found that participants in the mortality salience (vs. control) condition preferred the German options to foreign options.

Translating this to how mortality salience and worldview defense may impact attitudes and preferences for food, studies drawing on research probing the relationship between distress and the soothing qualities of food (e.g., Heatherton & Baumeister, 1991), found that consuming food (vs. not) mitigated worldview defense after mortality salience (Hirschberger & Ein‐Dor, 2005). Yet, studies indicate there may be nuances to this effect. For example, studies indicate that death reminders, coupled with a prime to consider a culturally valued healthy eater, led to the purchasing of more nutritious food items during a grocery store visit (McCabe et al., 2015). Studies also indicate that mortality salience decreased the consumption of high‐calorie foods for women with a high body mass index, presumably in an attempt to bolster one's self‐esteem by complying with societal norms of thinness (Goldenberg et al., 2005; see also Ferraro et al., 2005). Similarly, mortality salience (versus. a control topic) has been shown to lead participants to consume more of an ostensibly alcoholic beverage on the weekend (vs. a weekday), again, perhaps as a way to comply with socially delineated temporal scripts for alcohol consumption, that is, it is part of the cultural worldview to drink on the weekend versus a weekday (McCabe & Bartholow, 2019). Of relevance for the current research, studies also show that after mortality salience participants prefer a local, regional beer compared to foreign beer (Marchlewski, 2007), local soft drinks compared to foreign ones, and local chocolate compared to foreign ones. These preference shifts were even found to translate to actual consumption, with participants consuming more of the local product (relative to a foreign product) after mortality salience (Friese & Hofmann, 2008).

Despite considerable research probing the various manifestations of worldview defense after a mortality reminder, no research to date has examined how this might translate to safety perceptions in the domain of food. Inspired by patterns observed in takeaway food sales during the early stages of the COVID‐19 pandemic, we bring terror management theory to bear on this phenomenon which provides a unique opportunity to examine how foods from one's in‐group versus out‐group compare in terms of safety, and how experimentally manipulating death awareness might impact these food safety perceptions.

## 4 THE PRESENT RESEARCH

Motivated by contemporary news reports and changes in patterns of takeaway orders, the current research takes a terror management perspective to probe the implications of mortality reminders on evaluations of takeout food safety. Utilizing a mixed experimental design, U.S. participants were asked to rate the safety of American, Chinese, and Italian cuisines following a reminder of death used to induce mortality salience and the subsequent worldview defense (vs. a control topic and no worldview defense).

As noted, according to TMT, when death thoughts are active but outside of focal awareness, there are broadly two ways in which people defend their cultural worldview—either by increasing support for their own worldview and its associated symbols, beliefs, and products, or, by decreasing support for alternative worldviews and their associated symbol, beliefs, and products. Following this analysis, our central hypothesis posits that U.S. participants would rate American takeaway food as safer to consume following a reminder of death (vs. social exclusion) relative to foreign takeaway food. Importantly, Friese and Hoffman (2008) noted mixed evidence and ambiguities related to the manifestation of worldview defense. Specifically, results indicated that worldview defense evinced as food preferences tended to be driven by enhanced consumption of the in‐group's food (rather than decreased preference and consumption of the out‐group food). Further, on the point of ingroup versus outgroup manifestations of worldview defense; prior research suggests that when people across cultures are faced with a common global threat (such as is the case with the COVID‐19 pandemic), the outgroup derogation resulting from mortality salience may be less prone to occur due—while the championing of in‐group values and products remains (see, for example, Pyszczynski et al., 2012). Despite the potential for outgroup derogation to not manifest (or manifest less prominently), we opted to predict it would be expressed as lower evaluations of Chinese and Italian food safety following mortality salience (vs. control). We reasoned this was consistent with the broader pattern of findings in TMT research, and that there were significantly more studies demonstrating the outgroup derogation effect (vs. not). However, we also acknowledged mixed evidence and potential nuances related to this prediction in the context of food, and further ambiguities given the unique circumstances of the pandemic. We return to this point in the general discussion.

## 5 METHOD

### 5.1 Participants

Sample size calculations were informed by analyses of effect sizes from studies examining the influence of mortality salience on food‐related attitudes and behavior. These were consulted to anticipate the sample sizes necessary to achieve a sufficient level of power (0.80) to detect mortality salience effects within each condition, should such effects be present. These studies tended to range between having small to medium effect sizes (*d* = 0.3 to 0.5). The power analysis (G*Power; Faul et al., 2007), assuming *d* = 0.3, prescribed a total sample size of 236 participants. In case the current effect was smaller than that suggested, and to accommodate for potential attrition and exclusion criteria, we decided to aim for more than double this amount and collect data from approximately 500 participants.

Data were collected from 506 U.S. participants in early July 2020. The study was posted on Amazon Mechanical Turk—a crowd‐sourced internet workforce. The study was listed on the site as “an investigation into personality and attitudes.” After data cleaning, 488 participants remained (age:*M* = 37.57, *SD* = 12.32; 212 males; 272 female; 4 not reported). Participants were removed based on two criteria. First, being an outlier when considering their overall completion time, and second, examining the amount of engagement with the mortality salience/social exclusion writing task. Participants who wrote fewer than five words in these open‐ended questions were excluded from the final sample.^1^ Participants were compensated 50 cents for participation (completion time *M* = 810 s, *SD* = 312).

### 5.2 Materials and procedure

#### 5.2.1 Mortality salience

As in numerous previous studies (see Burke et al., 2010), those in the mortality salience condition responded to two open‐ended questions presented to the participant as a recently designed way to assess personality: “What do you think will happen to you when you die?” and “What do you think happens to you as you physically die.” Following previous research (e.g., Vess et al., 2009), those assigned to the control condition responded to parallel questions about social exclusion. Social exclusion was selected as the control topic for two reasons. First, because it has been used as a negative but non‐death‐related threat in prior research (thus keeping the valence of the manipulation the same, while differing on the mortality content), and second, because many people were socially isolating at this time and as such the topic represented a suitable reflection of a real‐world threat people may have currently been experiencing (or threatened with) during the pandemic (we return to this in the general discussion).

#### 5.2.2 Distraction task

Participants then completed a set of filler tasks designed to distract them from conscious thoughts of mortality. The tasks were included as prior research indicates worldview defense tends only to emerge when death thoughts are active but outside of focal awareness (see e.g., Pyszczynski et al., 1999). Participants first completed the Positive and Negative Affects Schedule (PANAS; Watson et al., 1988), a psychometric scale used commonly as distraction tasks in TMT research (see Steinman & Updegraff, 2015), after the mortality salience manipulation. It involves asking participants to indicate to what extent they are currently experiencing 24 positive and negative feelings and emotions on a 5‐point Likert scale (*1—very slightly (or not at all), 5—extremely*). After the PANAS, participants completed a series of additional filler questions similar to those used in extant TMT studies. In total, the delay tasks lasted *M* = 175.30 s, *SD* = 90.89.

#### 5.2.3 Food safety perceptions

To gauge perceptions of how safe foods were to consume, participants were asked to rate how safe they believed cuisine from the United States, China, and Italy were. Instructions read “We would like to know your opinions about food consumption. Please complete the items below.” The 5‐point Likert type questions asked “How safe do you think it is to consume the following takeaway meals?” (*1—very unsafe, 5—very safe*) with the types of food being presented in random order.

#### 5.2.4 Demographics

Participants then completed demographic information, including age, sex, employment status, income, education, and whether they order takeout food (*yes*/*no*).

## 6 RESULTS

### 6.1 Affect

A one‐way (death versus. social exclusion) ANOVA was conducted to probe the potential influence of mortality salience on affect. No effects emerged on positive affect (*α* = 0.91), *F*(1, 486) = 0.02, *p* = .903, or negative affect (*α* = 0.85), *F*(1, 486) = 0.49, *p* = .483.

### 6.2 Food safety perceptions

A 2 (between factor; death vs. social exclusion) × 3 (within factor: American vs. Chinese vs. Italian takeaway) mixed analysis of variance (ANOVA) was conducted. Assumptions were met for the between factor equality of variance, *F*(1, 486)'s < 2.47, *p*'s > .117. However, Mauchly's test of sphericity was significant, *χ* ^2^[2] = 42.22, *p* < .001. As such, within‐subjects effects reported below utilize the Greenhouse‐Geisser correction to account for the violation of this assumption.

There was a significant main effect of food type on food safety perceptions, *F*(2, 897) = 44.80, *p* < .001, *η*_p_^2^ = 0.08. Safety scores for the American food (*M* = 4.09) were higher compared to both the Chinese food (*M* = 3.89), *t*(487) = 5.60, *p* < .001., *d* = 0.51, and Italian food (*M* = 3.77), *t*(487) = 8.32, *p* < .001, *d* = 0.75. Chinese food was seen as safer than Italian food, *t*(487) = 4.17, *p* < .001, *d* = 0.38, but less safe than American food, *t*(487) = −5.60, *p* < .001, *d* = −5.1. Finally, Italian food was seen as less safe than both Chinese, *t*(487) = −4.17, *p* < .001, *d* = −0.37, and American food, *t*(487) = −8.32, *p* < .001, *d* = −0.75. There was no main effect of the mortality salience manipulation, *F*(1, 486) = 0.67, *p* = .414, *η*_p_^2^ < 0.01.

**FIGURE 1:**
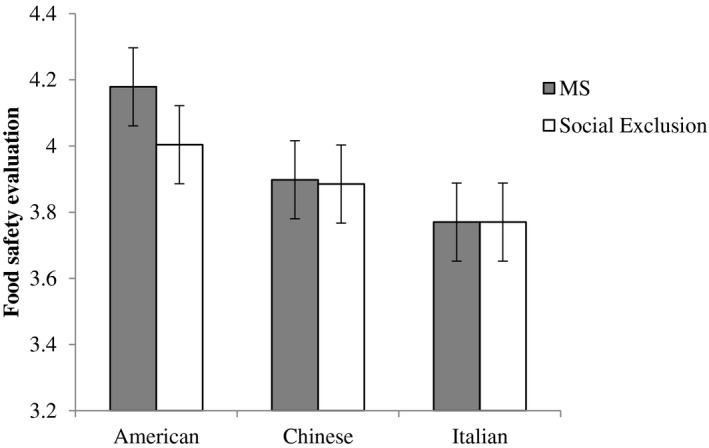
Two‐way interaction between MS and food type on safety evaluations (error bars represent 95% CIs)

As seen in Figure 1, as predicted, there was an interaction between food type and the mortality salience manipulation, *F*(2, 972) = 4.14, *p* = .016, *η*_p_^2^ = 0.01. American food was seen as safer after mortality salience compared to social exclusion, *t*(486) = 2.15, *p* = .032, *d* = 0.19. However, there was no difference in food safety perceptions for Chinese and Italian foods after mortality salience compared to social exclusion, *t*(486) = 0.15, *p* = .881, *d* = 0.01, and *t*(486) = −0.05, *p* = .957, *d* < 0.01, respectively.^2^

## 7 DISCUSSION

The present research suggests that reminders of mortality can lead to food from one's own culture being perceived as safer to consume compared to that of other cultures. This finding aligns with extant work highlighting heightened preference and consumption of food products from one's own culture following the activation of existential concerns and extends our knowledge by demonstrating this translates to the previously unexplored domain of food safety perceptions.

Our hypotheses were partially supported—when prompted by mortality salience, U.S. participants perceived American takeaway foods as safer to consume relative to Chinese or Italian takeout. However, while across conditions, the Italian and Chinese takeaway foods were rated as less safe relative to the American option, there was no evidence of worldview defense being expressed as lower food safety perceptions for the Italian or Chinese foods following the mortality salience manipulation (counter to our prediction).

The current results may help inform the psychological motivations influencing the patterns of consumer choice behavior noted during the early stages of the COVID‐19 outbreak. Specifically, while data from various news sources suggested a general trend of increased takeaway food consumption during the pandemic, this was not demonstrated for certain cuisines, namely Chinese and Italian takeout. The present study is the first to highlight the potential impact of existential concerns on consumers’ perceptions of food safety, which might then influence their takeaway food choice. As noted, it was hypothesized that MS would lead to heightened safety perceptions for the U.S. food, and lower safety perceptions for the Italian and Chinese foods. While the former aspect of the prediction held, the latter failed to manifest in our data. Despite this, we argue that the present research offers novel insight that helps inform the patterns of consumption observed during the pandemic. One possibility that might be considered, given results, is that takeout decisions may have been driven by people opting for American takeout food (over alternatives) as it is seen as safer to consume when existential concerns were looming large (likely a common experience during the pandemic; see Menzies & Menzies, 2020). Following TMT, these increased safety perceptions would be argued to be driven by the motivation to defend against mortality‐related cognition by bolstering the valuation of products from one's own culture, to reinstate existential security. In this way, the present research might suggest that the decreased interest in food from other cultures might not be driven by a decrease in safety perceptions induced by mortality reminders, but rather an increase in food safety perceptions of the U.S. food with implications for people switching where they would opt to order takeout from. Unfortunately, the present study cannot directly inform this potential switching behavior, and these are speculative implications ripe for further study and investigation.

Moving from the applied insight and potential implications to the consideration of methodological and conceptual factors, the lack of differences in safety perceptions of Chinese and Italian food following MS may appear somewhat inconsistent with the TMT idea of worldview defense. Specifically, the idea that such defense can manifest as both a supporting for of one's own culture (e.g., Rosenblatt et al., Study 3), but also as a derogation of others cultures (e.g., Rosenblatt et al., Study 1). One possible explanation for the lack of differences in the mortality salience (vs. control) conditions when judging the safety of Italian and Chinese takeaway might relate to common threats, such as a global pandemic, promoting a uniting of people and mitigating perceived differences/expressions of intergroup prejudices. Previous research supports this idea. For example, as climate change (e.g., global warming) was overwhelmingly viewed as a global problem, thinking about the universal threat of climate change should help foster a sense of “common humanity” and thus direct terror management efforts toward more prosocial, cooperative, and peaceful attitudes (an idea evidenced across three studies; see Pyszczynski et al., 2012). Further, recent evidence suggests that COVID‐19 can trigger mortality salience, and that this can, in some contexts, motivate more prosocial attitudes (Hu et al., 2020). While our study cannot speak to these ideas, it remains possible the pandemic is seen as a common threat, and thus the typical out‐group derogation as a function of MS did not emerge. As such, it is possible that future research, conducted during a period when there is not a global pandemic, or when the countries examined here are less saliently connected to the virus may yield different results. Indeed, prior studies conducted when there was not a global pandemic suggest death reminders do lead to reduced preferences for outgroup food (Friese & Hoffman, 2008).

On the topic of the pandemic, psychologists have long noted the importance of considering historical‐contextual factors in social psychological phenomenon, research, processes, study designs, and findings (e.g., Gergen, 1973; Sullivan, 2020). This is particularly critical given the present research was conducted during the early stages of the COVID‐19 pandemic (at least in the U.S.’s timeline). It is important that the current study and associated results are understood in the chronological context in at least two ways. First, in terms of considering study design choices. The control topic of social exclusion was selected for its mapping onto a real‐world threat that many were experiencing, or might experience, during the period. Over the course of data collection for this study (early July 2020), many countries had implemented a lockdown, so too had many United States—as such we believed it represented a more ecologically valid threat. In addition to reflecting a real threat many might face at this time, we also opted to use social exclusion as a control topic given it has frequently been used before in TMT research (see, for example, Vess et al., 2009) and (usually) constitutes a negative threat, albeit typically does not elicit concerns about mortality. As such, TMT studies employing social exclusion as a control topic can usually clearly compare measures following a task that involves eliciting the psychological threat of mortality versus a threat that does not. In this way, social exclusion versus death typically provides an appropriate test of one of the central ideas, that is, that worldview defense comes online specifically as a result of the existential threat of death, and not as a response to negative cognition or threats per se. However, given the lockdown put in place in many states following the outbreak of the virus, writing about social exclusion may have led some people to consider the virus, and by proxy, mortality. In this way, the study might have benefitted from using a “cleaner” threat as a control condition. As such, while there is a benefit to using a real‐world threat that people might have been experiencing (and thus bolstered the ecological validity of the study), there remains the possibility that the control topic harbored the capacity to activate mortality‐related cognition (perhaps more so for some than others, e.g., those in lockdown, the elderly who are shielding). Regardless of this potential limitation, differences in the in‐group food safety perceptions did emerge after MS (vs. control) in the current study, suggesting that these topics did elicit (at least to some extent) divergent psychological responses. The other way in which context influenced the design of this study was the choice to focus on Chinese and Italian takeaway as part of the dependent measures. This choice was motivated by the focus at the time on these countries that made them salient due to high levels of COVID‐19 infections. Since earlier in the year, the focus has shifted to different countries as infection peaks, and death rates shifts. As such, findings reported here could be, at least in part, a product of geographic factors and infection/mortality rates at the time.

The second point about context relates to the often rapidly changing social landscape during the time of the pandemic. As such, it is important that we locate social psychological research in its appropriate epoch and understand that many of the “conventional” patterns of behavior typically observed were disrupted during this period. Consequently, as previously discussed, the downstream effects typically observed in TMT studies as a result of mortality salience (such as out‐group derogation) may not manifest in these unprecedented times. Overall, there is much ambiguity and uncertainty about the generalizability of social psychological findings at this time, with distinct problems for TMT research. One of the difficulties with TMT research, at the moment, is that people are to a greater or lesser degree “living in the treatment condition.” Mortality concerns and reminders are likely higher than usual given the pandemic (Menzies & Menzies, 2020), and worldview defense may, for example, be more chronically activated or have muted activation due to existential threat becoming so commonplace and people acclimating or becoming numb to the bombardment of mortality reminders. How TMT processes operate in such a world is currently unclear, and more research will be needed to inform these uncertainties. The present study makes a small contribution to this area, by noting that differences emerged in perceptions of food safety for the American takeout option after mortality salience. In this way, we note that experimentally elevating death concerns, even during a time when existential concerns may be elevated generally, can still have an impact on people's food safety perceptions, and thus worldview defense may still emerge in some semblance. Further, effects reported might be larger than indicated given the control topic could potentially activate death concerns (as discussed, a trade‐off against using a more ecologically valid topic, i.e., social exclusion). In addition, we used a distraction task that was shorter than those found in other TMT studies. A meta‐analysis of TMT studies indicates longer distraction tasks tend to generate larger effects (Steinman & Updegraff, 2015). As such, this shorter task might also have undermined the potency of the MS induction.

Returning to the observed patterns of takeout food that inspired this study and noting the many ambiguities about conducting research during the time of COVID, the possibility remains, and there is some evidence from the present research, that the upturn in takeaway orders, but Italian and Chinese cuisine missing out, may be driven by concerns about mortality resulting in increased food safety perceptions of U.S. takeout. As such, people may have opted for U.S. takeout over alternatives due to food safety perceptions, resulting in less interest in Italian and Chinese food outlets, and these providers taking a financial hit. Further, while we did observe lower food safety scores for both Chinese and Italian takeaway across conditions, we have no evidence to suggest that food safety perceptions of the Chinese and Italian cuisines were impacted by temporarily heightened mortality cognition via the experimental manipulations used here. As such, the present study informs a real‐world phenomenon by noting that concerns around food safety reported in the media and the lower interest in takeout orders for Chinese and Italian food, may not be driven by explicit reminders of mortality prompting worldview defense—at least not as operationalized in the present study. However, the possibility remains that the differences in food safety perceptions that were observed across conditions are, indeed, influenced by existential concerns that we simply did not capture or instill with this particular study design. Future research might attempt to address this possibility by, for example, examining other existential influences such as death anxiety and existential isolation, and observing how they correlate against food safety perceptions for in‐group versus out‐group cuisine.

## 8 CONCLUSION

The present research builds on extant work, demonstrating heightened preference and consumption of food from one's own culture (vs. others) after mortality salience. We extend this work in a novel direction, demonstrating worldview defense following thoughts about mortality can manifest as increased perceptions of the safety of food from one's own culture. This may suggest concerns about mortality and efforts to manage the associated existential anxieties could, at least in part, contribute to the increased consumption of takeaway food from one's own culture as it is deemed “safer.” This study also informs the noted downturn in interest in Chinese and Italian food providers during the early stages of the COVID‐19 pandemic, as people perhaps opted for American alternatives due to them being seen as safer to consume. Ambiguities were also discussed, centering on the manifestations of worldview defense given there was no evidence mortality reminders led participants to view Chinese or Italian takeout as less safe. In sum, the COVID‐19 pandemic has impacted many aspects of our social world and behaviors. The present study offers a novel contribution to understanding this impact by extending our understanding of the influence of mortality concerns on food safety perceptions.

## Data Availability

Data related to this study will be made available publicly online during 2021. The corresponding author may be contacted for data related queries and requests before then.

## Footnotes

^1^
Recent research indicates data quality measures such as examining outliers on completion time may not be sufficient to detect poor quality responses when using online participant pools (e.g., Barends & De Vries, 2019). As such, the decision to remove those who wrote fewer than five words was taken based on examining prior data from similar writing tasks in TMT studies conducted by the first author. These data indicated participants typically wrote at least five words. Retaining the participants who wrote fewer than five words (*n* = 4) in the data set does not alter the pattern of results reported.

^2^
The pattern of results described here remains when controlling for whether participants indicated they order takeaway food or not.
